# KIF22 Promotes Development of Pancreatic Cancer by Regulating the MEK/ERK/P21 Signaling Axis

**DOI:** 10.1155/2022/6000925

**Published:** 2022-05-06

**Authors:** Ruiyun Zhang, Li Ma, Yucai Wei, Kongkong Wei, Tianliang Song, Zhixing Du, Zhijun Feng

**Affiliations:** ^1^Southern University of Science and Technology Hospital, No. 6019, Liuxian Avenue, Nanshan District, Shenzhen, Guangdong, China; ^2^Gansu Provincial Hospital, No. 204, Donggang West Road, Chengguan District, Lanzhou, Gansu, China; ^3^Lanzhou University Second Hospital, No. 82, Cuiyingmen, Chengguan District, Lanzhou, Gansu, China

## Abstract

The study is aimed at exploring the potential biological process and molecular mechanism of KIF22 involved in the development and progression of pancreatic cancer. First, we used the GEPIA database and tissue qRT-PCR to examine the expression of KIF22 mRNA in pancreatic cancer. Meanwhile, immunohistochemistry revealed the presence of KIF22 in 71 pancreatic cancer tissues versus 30 paracarcinoma tissues. Then, we also explored the relationship between KIF22 expression level and clinical prognosis. Furthermore, in pancreatic cancer cells, we silenced KIF22 by transfecting KIF22 SiRNA, and we investigated the effect of KIF22 on the proliferation of pancreatic cancer cells with MTT and colony formation assays. Finally, we used Gene Set Enrichment Analysis (GSEA) to look at the effect of KIF22 on the cell cycle regulation of pancreatic cancer cells, and we used Western blot to look at the relationship between KIF22 and the phosphorylated MEK1/2, ERK1/2 (p-MEK1/2, p-ERK1/2), and the cyclin-dependent kinase inhibitor (P21). In this study, we found that KIF22 was highly expressed in pancreatic cancer tissues, and patients with high expression of KIF22 demonstrated significantly worse clinical prognosis outcomes (*P* < 0.05). When the KIF22 gene was silenced in pancreatic cancer cells (PANC-1 and MIA PaCa-2), the cells' ability to proliferate was significantly reduced. Furthermore, GSEA confirmed that KIF22 is involved in cell cycle regulation in pancreatic cancer patients (FDR = 0.00158, *P* < 0.0001). Besides, the level of KIF22 expression was positively correlated with Ki67 (*r* = 0.8043, *P* < 0.0001), and KIF22 can promote the transmutation of G1/S. The expression of p-MEK1/2 and p-ERK1/2 was significantly downregulated, while P21 expression was significantly upregulated (*P* < 0.05). According to our findings, KIF22 is highly expressed in pancreatic cancer and demonstrates a poor clinical prognosis. It regulates the cell cycle via the MEK/ERK/P21 signaling axis and promotes the development of pancreatic cancer.

## 1. Introduction

Globally, pancreatic cancer ranks the seventh leading cause of cancer related-death [1]. In the United States in 2019, 45,750 deaths from pancreatic cancer occurred, and 56,770 new cases were found, with a five-year survival rate of only 9% [2]. For a long time, gemcitabine-based combination chemotherapy has been the main treatment for patients with locally advanced or metastatic pancreatic cancer, but because some patients exhibit primary or acquired resistance, which is a major barrier to treatment, the survival rate has not improved significantly [3, 4]. As a result, a thorough investigation of the molecular mechanism involved in the pathogenesis and progression of pancreatic cancer is particularly important for proposing new and effective strategies for the diagnosis and treatment of pancreatic cancer. The microtubule cytoskeleton is a well-established therapeutic target in cancer [5]. Interestingly, researchers reported that kinesins involved in mitosis play an important role in cancer development and drug resistance [6, 7], and inhibitors targeting KIF11 entered phase I and II clinical trials [8]. Therefore, screening new effective proteins from the KIF family as a molecular target for developing new cancer inhibitors is thought to be a promising strategy for pancreatic cancer treatment. Using bioinformatic analysis, we discovered that kinesin family member 22 (KIF22) is highly expressed in pancreatic cancer and that upregulation of KIF22 is associated with a poor prognosis in pancreatic cancer patients. As a result, we hypothesized that KIF22 may be involved in the occurrence and development of pancreatic cancer. Following that, we investigated the role of KIF22 in pancreatic cancer, investigated the effect of KIF22 on pancreatic cancer development, and explored the underlying molecular mechanisms.

## 2. Materials and Methods

### 2.1. Materials

#### 2.1.1. Data Collection

We collected 6 pairs of pancreatic cancer (confirmed by pathology) and fresh tissues adjacent to cancer (more than 2 cm from the cancer tissue) that were surgically removed from the General Surgery Department of Second Hospital of Lanzhou University (hereinafter referred to as our department), preserved in liquid nitrogen, and used for mRNA extraction. From 2012 to 2015, 71 cases of pancreatic cancer tissue and 30 cases of paracancer tissue surgical specimens (confirmed by pathology) from our department were selected, embedded, and fixed in paraffin, immunohistochemically stained of pathological sections, corresponding pathological data were collected, and a 3-year follow up was performed. All patients presented with no history of radiotherapy and chemotherapy before surgery. Ethical approval was granted by the Lanzhou University Second Hospital Ethics Committee.

#### 2.1.2. Cells and Experimental Reagents

Pancreatic cancer cell lines PANC-1 and MIA PaCa-2 were purchased from the Shanghai and Kunming Cell Banks of the Chinese Academy of Sciences. The KIF22 primers used were purchased from Beijing Kinco Biotechnology Co., Ltd., fetal bovine serum was obtained from PEAK, United States, and Dulbecco's modified Eagle medium (DMEM) was purchased from Gibco, United States; MTT cell proliferation kit (colorimetric method) was purchased from BioVision, United States; KIF22 SiRNA and supporting transfection reagents were obtained from Shanghai Jima Pharmaceutical Company, PI staining solution used for cell cycle detection was purchased from Beijing Soleibao Company, and p-MEK1/2 (phosphorylated mitogen-activated protein kinase kinase, p-MAPKK; catalogue number: 9154) was purchased from Cell Signaling Technology in the United States, p-ERK1/2 (phosphorylated extracellular regulated kinase 1/2; catalogue number: 28733-1-AP), and P21 (cyclin-dependent kinase inhibitor 1A, CDKN1A; catalogue number: 10355-1-AP) antibodies were purchased from Proteintech Company in the United States.

### 2.2. Methods

#### 2.2.1. Analysis of KIF22 Expression Levels for Pancreatic Cancer

KIF22 expression levels for pancreatic cancer were assessed at both mRNA and protein levels. We validated the expression level of KIF22 in pancreatic cancer and normal samples using the online database Gene Expression Profiling Interactive Analysis (GEPIA) [9] (http://gepia.cancer-pku.cn/index.html). Furthermore, quantitative real-time PCR (qRT-PCR) was used to detect the KIF22 expression in pancreatic cancer and adjacent tissues, as shown below. Trizol method was used to extract total mRNA from 6 pairs of tissues, followed by reverse transcription and cDNA concentration detection. qRT-PCR was performed with GAPDH as an internal reference, and the relative expression level of KIF22 in each sample was calculated using the double *Δ*Ct method. For protein level, we investigated the KIF22 protein expression in pancreatic cancer and normal tissue sections by immunohistochemistry (IHC), as described in Kang et al. [10]. Two independent pathologists reviewed and scored the slices blindly, and the brown-yellow staining was positive. Staining score = staining intensity∗staining positive area; staining intensity score includes 0 points (negative staining), 1 point (weak staining), and 2 points (medium staining), 3 points (strong staining); staining positive area is based on the number of positive cells. Percentage score: 0 points (<5%), 1 point (5%~25%), 2 points (26%~50%), 3 points (51%~75%), and 4 points (>75%); staining score < 6 is divided into a low expression of KIF22, score ≥ 6 is divided into a high expression of KIF22 [11]. We also looked at possible links between KIF22 expression levels and clinicopathological features of pancreatic cancer during this session. Finally, we used survival analysis to investigate the effect of KIF22 expression on patient prognostic survival. Based on the results of the survival analysis, we divided the patients into two groups: those with high KIF22 expression and those with low KIF22 expression, based on the value of each patient's prognosis index.

#### 2.2.2. Cell Culture and Establishment of KIF22 Knockdown Cell Line

Panc-1 and MIA PACA-2 cells were inoculated in cell culture vials in a DMEM containing 10% fetal bovine serum. The cells were cultured at 37°C in 5% CO_2_ incubator, digested, and counted at the logarithmic growth stage, inoculated in 5∗10^5^ cells/well in 6-well plates, and cultured at 37°C in 5% CO_2_ incubator. When the convergence degree of cells reached 50%–70%, SiRNA1, SiRNA2, and negative control SiRNA were transfected into different wells, respectively (Text s1). After 48 h, the cell protein was measured with Western blotting, and the SiRNA with the best silencing effect was chosen as the effective interference sequence (KIF22 SiRNA) for subsequent experiments. All of the experiments were performed for at least three biological repeats.

#### 2.2.3. The Effect of KIF22 on Cell Proliferation Ability

MTT: the cells were transfected with KIF22 SiRNA and negative control SiRNA. After 48 hours, transfected cells were digested with trypsin and counted, diluted to 1∗10^4^ cells/ml. After thorough mixing, 200 *μ*l of cell suspension was added to each well of six 96-well plates, and MTT was added at 0 h, 24 h, 48 h, 72 h, 96 h, and 120 h after cell adhesion. The OD490nm value was measured 45 min after incubation.

Colony formation experiment: the above cells with a density of 1∗10^4^ cells/ml were diluted to 500 cells/ml, and 2 ml cell suspension per well was added to a 35 mm petri dish. When more than 80 cells were found in a single colony, the cells were fixed and stained.

#### 2.2.4. Cell Cycle Detection by Flow Cytometry

First, to investigate the mechanism by which KIF22 promotes the occurrence and progression of pancreatic cancer, we analyzed the downstream pathways affected by KIF22 through GSEA in the LinkedOmics (http://www.linkedomics.org) database [12] as follows. We chose RNA-Seq data from patient with pancreatic cancer from the database, and then, we performed GSEA-KEGG pathway analysis using the Pearson card square method.

After the cells were transfected with SiRNA for 48 hours, the cells were collected by centrifugation at 1000 rpm for 5 minutes, fixed with 75% ethanol at 4°C for 2 hours, labeled with PI staining solution, and detected by flow cytometry.

#### 2.2.5. Molecules Involved in Downstream Signaling Regulation of KIF22 Were Examined Using Western Blot Analysis

Molecules involved in downstream signaling regulation of KIF22 were examined using the Western blot and Pearson's correlation test. The expressions of KIF22, p-MEK1/2, p-ERK1/2, and P21 cells were detected using Western blot; 48 h after SiRNA transfection, the total protein in 1∗10^6^ cells was extracted with 130 *μ*l lysis buffer. Then, the membrane was washed with Tris-Buffered Saline with Tween (TBST) and incubated the horseradish peroxidase-labeled secondary antibody (1 : 12,000) with a shaker at room temperature for 2 h. The membrane was washed with TBST once more, the protein band was scanned with the MiniChemi chemiluminescence system, and the gray value of the band was analyzed with ImageJ.

### 2.3. Statistical Analysis

SPSS 23.0 was used to analyze pathological data and experimental data, and GraphPad Prism 8 was used to plot. The measurement data demonstrated a normal distribution and were expressed as mean ± standard deviation. Two independent sample *T*-tests were used to compare the independent sample and paired data. The count data were expressed as rates and compared between groups by *χ*^2^ test. Linear regression was used to calculate the correlation between the two variables. The Kaplan-Meier method was used for survival analysis. *P* < 0.05 was considered statistically significant.

## 3. Results

### 3.1. High Expression of KIF22 Is a Biological Label of Worse Prognosis for Pancreatic Cancer Patients

**Figure 1:**
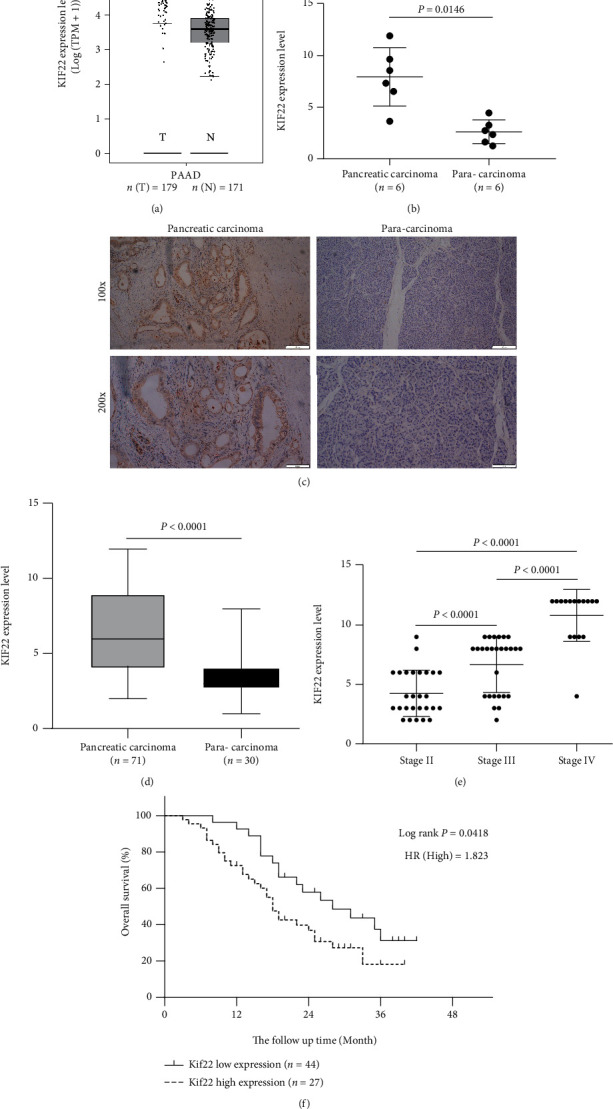
KIF22 is highly expressed in pancreatic cancer and marks the worse clinical prognosis. (a) KIF22 mRNA expression levels in pancreatic adenocarcinoma (PAAD) tissues (*T* = 179) and normal tissues (*N* = 171) analyzed by Gene Expression Profiling Interactive Analysis (GEPIA). ^∗^*P* < 0.05. (b) The mRNA expression levels of KIF22 in pancreatic carcinoma tissues and paracarcinoma tissues. (c, d) KIF22 protein levels in pancreatic carcinoma tissues are significantly higher than those in paracarcinoma tissues. (e) The level of KIF22 expression is related to the clinical pathological stage. (f) Patients with high KIF22 exhibit a lower overall survival rate.

On the mRNA level, the results from the GEPIA database (Figure 1(a)) and the fresh tissue qRT-PCR (Figure 1(b)) showed the following. KIF22 is highly expressed in pancreatic cancer. On the protein level, immunohistochemical results (Table s1) revealed that the staining intensity and positive area of KIF22 in cancer tissues were stronger than those in adjacent tissues (Figure 1(c)), and the staining score of cancer tissues was higher and statistically significant (*P* < 0.05, Figure 1(d)). The KIF22 expression level was also higher in patients with the higher pathological stage (Figure 1(e)).

Furthermore, patients with high KIF22 expression demonstrated a significantly lower overall survival rate than patients with low KIF22 expression (Figure 1(f), *P* < 0.05). Meanwhile, we divided our 71 patients into two groups based on the value of each patient's prognosis index. The high-KIF22 expression group exhibited 44 cases, and the low-KIF22 expression group exhibited 27 cases. The results of the association between levels of KIF22 protein expression and clinicopathological features revealed that a high level of KIF22 expression is associated with increased serum CA199 levels, a high Ki67 proliferation index, and a high stage, and these differences were statistically significant (Table 1, *P* < 0.05), but no statistically significant difference was found between the two groups in terms of age, gender, pathological type, differentiation, and status of lymph node metastasis (Table 1, *P* > 0.05).

### 3.2. Silencing KIF22 Inhibits the Proliferation of Pancreatic Cancer Cells

**Figure 2:**
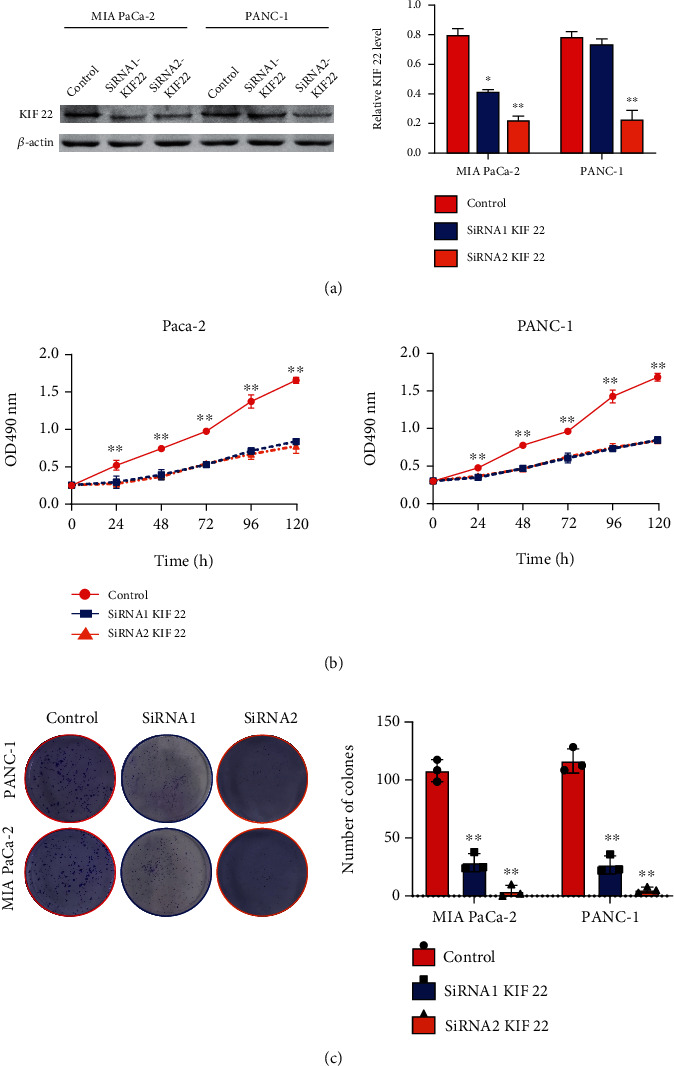
The proliferation ability of pancreatic cancer cells was reduced by silenced KIF22 in PANC-1 and MIA PaCa-2. (a) Effective knockdown of KIF22 expression in cells could be achieved via SiRNA2. (b) Silencing KIF22 reduces the proliferation rate of pancreatic cancer cells via MTT assay. (c) Silencing KIF22 reduces the colony forming ability of pancreatic cancer cells by the colony forming assay.

Western blot verified that SiRNA1 and SiRNA2 are effective interference sequences (Figure 2(a)), which can be used in subsequent experiments. MTT and colony formation experiments revealed that silencing the expression of KIF22 in pancreatic cancer cells significantly reduced cell proliferation ability, including decreased proliferation rate (Figure 2(b)) and decreased cell colony formation ability (Figure 2(c)).

### 3.3. KIF22 Is Involved in the Cycle Regulation of Pancreatic Cancer Cells

**Figure 3:**
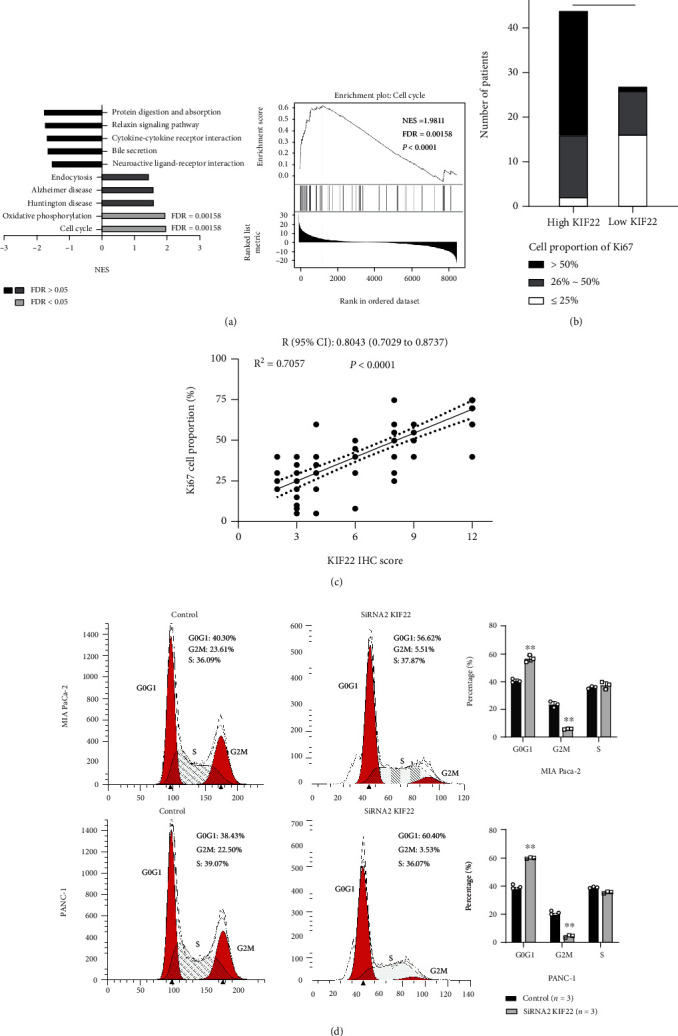
KIF22 plays a vital role in the regulation of the pancreatic cancer cell cycle. (a) KIF22 is highly enriched in the cell cycle pathway in pancreatic cancer analyzed by Gene Set Enrichment Analysis (GSEA). (b, c) The level of KIF22 expression was found to be positively correlated with the positive ratio of Ki67 cells. (d) Silencing KIF22 causes the significant upregulation of G1 and S phase populations in PANC-1 and MIA-PaCa-2, as well as downregulation of G2 and M phase populations.

To explore the mechanism of KIF22 promoting the occurrence and development of pancreatic cancer, we analyzed the pathway of KIF22 enrichment through GSEA and found that it was highly enriched in the cell cycle regulation pathway (Figure 3(a)). Clinical samples confirmed that the proportion of Ki67-positive staining cells in patients with high KIF22 expression was significantly higher than in patients with low KIF22 expression (Figure 3(b)), and the proportion of Ki67-positive cells was positively correlated with KIF22 expression level (Figure 3(c), *r* = 0.8043, *P* < 0.0001). Subsequently, we selected SiRNA2, which demonstrates the strongest inhibitory effect, for mechanism experiments and flow cytometry detection. The results also showed that after silencing KIF22 expression in pancreatic cancer cells, the proportion of cells in the G2/M phase decreased significantly, while the proportion of cells in the G1/S phase increased significantly, inducing G1/S phase arrest (Figure 3(d), *P* < 0.05).

### 3.4. KIF22 Is Involved in the Regulation of the MEK/ERK/P21 Signaling Pathway

**Figure 4:**
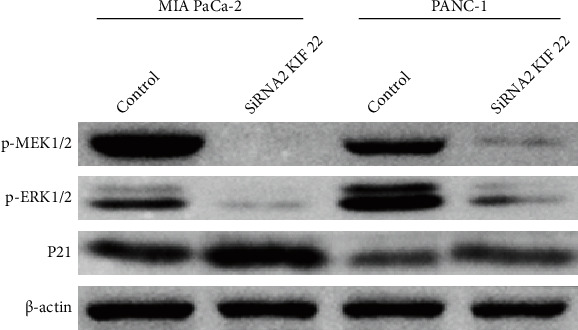
Silencing KIF22 downregulates p-MEK1/2 and p-ERK1/2 and upregulates P21 expression.

Western blot confirmed that silencing intracellular KIF22 expression significantly downregulated phosphorylated MEK1/2 and ERK1/2 (P-MEk1/2, P-ERk1/2) and upregulated cyclin-dependent kinase inhibitor P21 (*P* < 0.05, Figure 4).

## 4. Discussion

Pancreatic cancer is the most malignant tumor in the digestive system tumors. At the moment, surgical radical resection is the most effective method of treating pancreatic cancer. However, due to a lack of effective early detection, only 5%~10% of patients can be radically resected. Furthermore, due to insensitivity to radiotherapy and chemotherapy, the adjuvant treatment effect is not good, and the postoperative recurrence rate is extremely high. KIF22 is a member of the kinesin family and is involved in spindle formation during mitosis [13, 14]. KIF22 is abnormally expressed in cervical cancer, ovarian cancer, breast cancer, lung cancer, and prostate cancer [13, 15]. It can also reduce the transcriptional activity of CDC25C, delay the mitosis exit, and activate the TGF-*β*/EMT pathway to promote the proliferation and metastasis of breast cancer cells [13]. Furthermore, researchers reported that KIF22 can influence the biological processes of phosphorylation for epidermal growth factor receptor (EGFR), which can weaken EGFR internalization and promote EGF-dependent lung cancer cell proliferation [16]. However, no reports exist on the research of KIF22 in pancreatic cancer.

We discovered that KIF22 was abnormally overexpressed in pancreatic cancer tissue using the GEPIA database, qRT-PCR in fresh tissues, and immunohistochemical staining in pathological sections in this study. Furthermore, high expression of KIF22 was correlated with a high level of CA199 in serum, an advanced tumor stage, and an unfavorable prognosis in pancreatic cancer. Silencing KIF22 in pancreatic cancer cells significantly reduces cell proliferation ability, demonstrating that KIF22 can promote the occurrence and progression of pancreatic cancer. KIF22 is involved in the cycle regulation of pancreatic cancer cells, according to GSEA and verification. Clinical sample verification revealed that cell-proliferating nuclear antigen Ki67 expression levels were positively correlated with KIF22 expression levels. Further flow cytometry showed that silencing KIF22 could induce G1/S phase arrest. Western blot verified that after silencing KIF22, p-MEK1/2 and p-ERK1/2 were significantly downregulated, while P21 was significantly upregulated. It was finally proven that KIF22 can participate in the cycle regulation of pancreatic cancer cells via the MEK/ERK/P21 signaling axis, thereby promoting the occurrence and progression of pancreatic cancer.

KIF22 can bind to microtubules and DNA, and this binding effect is regulated by CDK1 [17, 18]. As a result, the regulation of the cell cycle by KIF22 may depend on the MEK/ERK signaling axis closest to CDKs and the CDK1 inhibitor P21. MEK/ERK, as a downstream signaling axis, participates in variety of cell life activities, including proliferation, motility, and differentiation [19], and it is closely linked to the occurrence and development of tumors. Additionally, it is currently an important target for tumor therapy [20, 21], and its inhibitors entered phase II clinical trials with fruitful results [22]. This study demonstrates that KIF22 promotes the occurrence and development of pancreatic cancer, and it confirms that its cancer-promoting effect is potentially related to the MEK/ERK/P21 signaling axis. As a result, KIF22-targeting inhibitors may be used as adjuncts to MEK-ERK inhibitors to increase drug sensitivity and reduce biotoxicity, slow pancreatic cancer progression, reduce postoperative recurrence rate, and improve pancreatic cancer patients' survival outcomes.

## 5. Conclusion

In summary, KIF22 expression levels are negatively correlated with pancreatic cancer clinical prognosis, and it participates in cell cycle regulation via MEK/ERK/P21, promotes the occurrence and development of pancreatic cancer, can be used as a prognostic factor for pancreatic cancer patients, and may be a potential target for pancreatic cancer adjuvant therapy. However, further research into its inhibitor design and drug effect is required.

## Figures and Tables

**Table 1:** The relationship between KIF22 expression and clinicopathological features in patients with pancreatic cancer.

Clinicopathological characteristics	KIF22 high expression (*n* = 44)	KIF22 low expression (*n* = 27)	*P* value
Age	57.61 ± 9.08	58.41 ± 10.05	0.733
Sex			
Male	29 (65.91%)	18 (66.67%)	0.848
Female	15 (34.09%)	9 (33.33%)	
CA 199	477.35 ± 338.12	94.28 ± 89.35	<0.0001
Ki67			<0.0001
≤25%	2 (4.55%)	16 (59.26%)	
26%~50%	14 (31.82%)	10 (37.04%)	
>50%	28 (63.64%)	1 (3.7%)	
Pathological type			
Ductal adenocarcinoma	25 (56.82%)	14 (51.85%)	0.683
Adenocarcinoma	19 (43.18%)	13 (41.15%)	
Differentiation			
Low differentiation	13 (29.55%)	10 (37.04%)	0.744
Moderate differentiation	26 (59.09%)	15 (55.56%)	
High differentiation	5 (11.36%)	2 (7.41%)	
AJCC pathological stage			
II	10 (22.73%)	17 (62.96%)	0.013
III	18 (40.91%)	9 (33.33%)	
IV	6 (36.36%)	1 (3.70%)	
T stage			
T1	2 (4.55%)	5 (18.52%)	0.001
T2	19 (43.18%)	20 (74.07%)	
T3	13 (29.55%)	1 (3.7%)	
T4	10 (22.73%)	1 (3.7%)	
N stage			
N0	13 (29.55%)	12 (44.44%)	0.05
N1	26 (59.09%)	10 (37.04%)	
N2	5 (11.36%)	5 (18.52%)	

## Data Availability

The data used to support the findings of this study are available from the corresponding author upon request.

## Supplementary Materials

Supplementary 1 Text s1: supplementary materials related to the operation of cell experiments.

Supplementary 2 Table s1: details of the clinical cases.docx.
