# Supplementary material for: KIF22 Promotes Development of Pancreatic Cancer by Regulating the MEK/ERK/P21 Signaling Axis
Source: Biomed Res Int. 2022 May 6;2022:6000925. doi: 10.1155/2022/6000925 (PMC9107036; doi:10.1155/2022/6000925)
Supplement: Supplementary 1 — Text s1: supplementary materials related to the operation of cell experiments. [file 6000925.f1.docx]

**Supplementary Materials**

**1.1 SiRNA sequences:**

SiRNA-1:

sense 5-GGUCCAAGGAGGUGAUCAATT-3,

antisense, 5-UUGAUCACCUCCUUGGACCTT-3;

SiRNA 2:

sense, 5-AGAGAAGGACCUAGAGAUUTT-3,

antisense, 5-AAUCUCUAGGUCCUUCUCUTT-3;
SiRNA 3:

sense, 5-CACCAGGAGACUCUCAAAUTT-3,
antisense, 5-AUUUGAGAGUCUCCUGGUGTT-3.

Control:
sense, 5-UUCUCCGAACGUGUCACGUTT-3，

antisense, 5-ACGUGACACGUUCGGAGAATT-3.

**1.2 Transfection method:**

⑴. One day before transfection, 4-5*10^4^ cells were inoculated on 24-well plates with 0.5ml DMEM containing FBS and antibiotics.

⑵. The number of cells selected for initial inoculation should be able to achieve 70-90% cell confluence within 24 hours.

⑶. Add 20pmol SiRNA to 50μl DMEM serum-free medium, mixed gently.

⑷. Mixed lipofectamine reagent, dilute 1μl lipofectamine reagent with 50μl serum-free DMEM, mixed gently, placed at room temperature for 5 minutes;

⑸. Mixed the diluted SiRNA and RNAi-mate reagent; Mixed gently and placed at room temperature for 20 minutes to form SiRNA/lipofectamine complexes.

⑹. Added 100μl SiRNA/lipofectamine complexes to the well of the culture plate containing cells and culture medium, and gently shake the cell culture plate back and forth.

⑺. If cell lines are sensitive, remove the complex and replace the medium after 6 hours of incubation.

⑻. The cells were incubated at 37℃ for 48h in a CO_2_ incubator, and other detection steps were performed after transfection.

**1.3 Protein extraction**

Eighteen hours after inoculation of cells, when the cell density reached 70-90%, washed with PBS, digested with trypsin, and centrifuged to collect cells. Add 30μl of lysis buffer (1M Tris-HCl (PH-7.4) 50μl, 1M NaCl 150μl, 0.5M EDTA 2μl, TritonX-100 20μl, H2O 778μL）) to 1*10^6^ cells, ice bath for 10min, and vortex every 5min to fully lyse. Then, sonicated the protein sample, and centrifuge at 12000g for 10min at 4℃. Take the supernatant (30μl) + 6μl Loading buffer (5X), boil the protein at 100 degrees for 5min, ice bath for 1min, centrifuge at 12000g for 5min, take 10μl to WB.
