# Supplementary material for: KIF22 Promotes Development of Pancreatic Cancer by Regulating the MEK/ERK/P21 Signaling Axis
Source: Biomed Res Int. 2022 May 6;2022:6000925. doi: 10.1155/2022/6000925 (PMC9107036; doi:10.1155/2022/6000925)
Supplement: Supplementary 2 — Table s1: details of the clinical cases.docx. [file 6000925.f2.docx]

| **Serial Number** | **Gender （male 1 female 2）** | **Age** | **Diagnosis** | **CA199（U/ml）** | **P-Stage** | **T** | **N** | **Stage** | **KI67** | **KIF22-IHC Score (Cancer Tissue)** | **Expressed （1High 2Low）** | **KIF22-IHC Score (Normal Tissue)** | **Survival time (Month)** | **Outcome (0 live 1 dead)** | **Tumor Classification** | **Differentiated Degree** |
| --- | --- | --- | --- | --- | --- | --- | --- | --- | --- | --- | --- | --- | --- | --- | --- | --- |
| **1** | **1** | **59** | **Pancreatic** **cancer** | **515.42** | **T3N0M0** | **3** | **0** | **Ⅲ** | **75** | **8** | **1** | **4** | 12 | 0 | **Infiltrating Ductal Adenocarcinoma** | **Poorly** |
| **2** | **2** | **66** | **Pancreatic cancer** | **1000.00** | **T2N0M0** | **2** | **0** | **Ⅱ** | **60** | **9** | **1** | **3** | 13 | 1 | **Infiltrating Ductal Adenocarcinoma** | **Moderately** |
| **3** | **1** | **47** | **Pancreatic cancer** | **219.70** | **T3N1M0** | **3** | **1** | **Ⅲ** | **55** | **8** | **1** | **6** | 17 | 1 | **Infiltrating Ductal Adenocarcinoma** | **Moderately** |
| **4** | **1** | **49** | **Pancreatic cancer** | **247.20** | **T2N1M0** | **2** | **1** | **Ⅲ** | **60** | **8** | **1** |  | 9 | 1 | **Adenocarcinoma** | **Moderately** |
| **5** | **1** | **53** | **Pancreatic cancer** | **456.00** | **T4N2M0** | **4** | **2** | **ⅣB** | **75** | **12** | **1** | **4** | 24 | 0 | **Adenocarcinoma** | **Moderately** |
| **6** | **1** | **59** | **Pancreatic cancer** | **361.90** | **T2N0M0** | **2** | **0** | **Ⅱ** | **45** | **6** | **1** | **6** | 30 | 0 | **Adenocarcinoma** | **Poorly** |
| **7** | **1** | **55** | **Pancreatic cancer** | **252.29** | **T3N0M0** | **3** | **0** | **Ⅲ** | **60** | **8** | **1** | **2** | 10 | 0 | **Infiltrating Ductal Adenocarcinoma** | **Moderately** |
| **8** | **2** | **52** | **Pancreatic cancer** | **338.23** | **T4N1M0** | **4** | **1** | **ⅣA** | **75** | **12** | **1** | **1** | 19 | 1 | **Adenocarcinoma** | **Moderately** |
| **9** | **1** | **62** | **Pancreatic cancer** | **273.00** | **T3N1M0** | **3** | **1** | **Ⅲ** | **60** | **9** | **1** | **3** | 7 | 1 | **Infiltrating Ductal Adenocarcinoma** | **Moderately** |
| **10** | **2** | **70** | **Pancreatic cancer** | **488.70** | **T4N0M0** | **4** | **0** | **ⅣA** | **55** | **9** | **1** |  | 40 | 0 | **Infiltrating Ductal Adenocarcinoma** | **Poorly** |
| **11** | **1** | **61** | **Pancreatic cancer** | **424.66** | **T1cN1M0** | **1** | **1** | **Ⅱ** | **8** | **6** | **1** |  | 31 | 1 | **Adenocarcinoma** | **Well** |
| **12** | **1** | **62** | **Pancreatic cancer** | **247.80** | **T2N1M0** | **2** | **1** | **Ⅲ** | **55** | **8** | **1** | **6** | 3 | 1 | **Infiltrating Ductal Adenocarcinoma** | **Poorly** |
| **13** | **1** | **48** | **Pancreatic cancer** | **1000.00** | **T4N1M0** | **4** | **1** | **ⅣA** | **70** | **12** | **1** | **3** | 18 | 1 | **Infiltrating Ductal Adenocarcinoma** | **Moderately** |
| **14** | **1** | **74** | **Pancreatic cancer** | **954.60** | **T3N0M0** | **3** | **0** | **Ⅲ** | **60** | **8** | **1** | **8** | 7 | 1 | **Infiltrating Ductal Adenocarcinoma** | **Poorly** |
| **15** | **2** | **48** | **Pancreatic cancer** | **134.33** | **T4N1M1** | **4** | **1** | **ⅣB** | **70** | **12** | **1** |  | 25 | 1 | **Infiltrating Ductal Adenocarcinoma** | **Poorly** |
| **16** | **1** | **63** | **Pancreatic cancer** | **228.80** | **T2N0M0** | **2** | **0** | **Ⅱ** | **50** | **6** | **1** |  | 11 | 1 | **Infiltrating Ductal Adenocarcinoma** | **Moderately** |
| **17** | **1** | **67** | **Pancreatic cancer** | **626.90** | **T3N1M1** | **3** | **1** | **ⅣB** | **75** | **12** | **1** |  | 18 | 1 | **Adenocarcinoma** | **Poorly** |
| **18** | **1** | **58** | **Pancreatic cancer** | **217.40** | **T2N1M0** | **2** | **1** | **Ⅲ** | **60** | **9** | **1** |  | 25 | 1 | **Adenocarcinoma** | **Moderately** |
| **19** | **1** | **53** | **Pancreatic cancer** | **145.40** | **T2N0M0** | **2** | **0** | **Ⅱ** | **60** | **8** | **1** | **2** | 10 | 1 | **Infiltrating Ductal Adenocarcinoma** | **Moderately** |
| **20** | **2** | **52** | **Pancreatic cancer** | **818.80** | **T3N1M1** | **3** | **1** | **ⅣB** | **60** | **12** | **1** |  | 20 | 0 | **Adenocarcinoma** | **Moderately** |
| **21** | **1** | **65** | **Pancreatic cancer** | **65.97** | **T2cN0M0** | **2** | **0** | **Ⅱ** | **50** | **6** | **1** | **3** | 19 | 1 | **Infiltrating Ductal Adenocarcinoma** | **Moderately** |
| **22** | **2** | **47** | **Pancreatic cancer** | **127.10** | **T2N1M0** | **2** | **1** | **Ⅲ** | **40** | **8** | **1** | **6** | 6 | 1 | **Infiltrating Ductal Adenocarcinoma** | **Moderately** |
| **23** | **1** | **59** | **Pancreatic cancer** | **1000.00** | **T3N2M1** | **3** | **2** | **ⅣB** | **40** | **12** | **1** |  | 36 | 0 | **Adenocarcinoma** | **Poorly** |
| **24** | **1** | **52** | **Pancreatic cancer** | **725.70** | **T1cN1M0** | **1** | **1** | **Ⅱ** | **50** | **6** | **1** | **4** | 4 | 1 | **Adenocarcinoma** | **Well** |
| **25** | **1** | **38** | **Pancreatic cancer** | **967.90** | **T3N1M1** | **3** | **1** | **ⅣB** | **70** | **12** | **1** | **2** | 13 | 1 | **Infiltrating Ductal Adenocarcinoma** | **Moderately** |
| **26** | **2** | **57** | **Pancreatic cancer** | **1000.00** | **T3N2M0** | **3** | **2** | **ⅣA** | **75** | **12** | **1** |  | 9 | 1 | **Infiltrating Ductal Adenocarcinoma** | **Moderately** |
| **27** | **1** | **65** | **Pancreatic cancer** | **295.20** | **T4N1M1** | **4** | **1** | **ⅣB** | **70** | **12** | **1** |  | 15 | 1 | **Adenocarcinoma** | **Poorly** |
| **28** | **1** | **61** | **Pancreatic cancer** | **235.90** | **T4N1M0** | **4** | **1** | **ⅣA** | **40** | **9** | **1** |  | 17 | 1 | **Adenocarcinoma** | **Moderately** |
| **29** | **2** | **53** | **Pancreatic cancer** | **596.20** | **T2N0M0** | **2** | **0** | **Ⅱ** | **50** | **6** | **1** |  | 29 | 0 | **Infiltrating Ductal Adenocarcinoma** | **Moderately** |
| **30** | **1** | **69** | **Pancreatic cancer** | **564.10** | **T2N1M0** | **2** | **1** | **Ⅲ** | **50** | **9** | **1** |  | 19 | 1 | **Infiltrating Ductal Adenocarcinoma** | **Poorly** |
| **31** | **1** | **63** | **Pancreatic cancer** | **40.06** | **T2N1M0** | **2** | **1** | **Ⅲ** | **30** | **8** | **1** |  | 18 | 1 | **Infiltrating Ductal Adenocarcinoma** | **Well** |
| **32** | **1** | **50** | **Pancreatic cancer** | **579.60** | **T2N1M0** | **2** | **1** | **Ⅲ** | **55** | **9** | **1** |  | 28 | 1 | **Infiltrating Ductal Adenocarcinoma** | **Moderately** |
| **33** | **2** | **44** | **Pancreatic cancer** | **198.20** | **T3N1M0** | **3** | **1** | **Ⅲ** | **55** | **9** | **1** |  | 30 | 0 | **Infiltrating Ductal Adenocarcinoma** | **Moderately** |
| **34** | **1** | **46** | **Pancreatic cancer** | **170.70** | **T2N0M0** | **2** | **0** | **Ⅱ** | **50** | **6** | **1** |  | 30 | 0 | **Infiltrating Ductal Adenocarcinoma** | **Well** |
| **35** | **1** | **59** | **Pancreatic cancer** | **98.98** | **T2N0M0** | **2** | **0** | **Ⅱ** | **30** | **6** | **1** |  | 8 | 1 | **Infiltrating Ductal Adenocarcinoma** | **Moderately** |
| **36** | **2** | **66** | **Pancreatic cancer** | **1000.00** | **T4N2M1** | **4** | **2** | **ⅣB** | **75** | **12** | **1** |  | 24 | 1 | **Adenocarcinoma** | **Moderately** |
| **37** | **2** | **50** | **Pancreatic cancer** | **306.70** | **T3N1M0** | **3** | **1** | **Ⅲ** | **60** | **8** | **1** |  | 10 | 1 | **Adenocarcinoma** | **Moderately** |
| **38** | **2** | **45** | **Pancreatic cancer** | **1000.00** | **T4N1M0** | **4** | **1** | **ⅣA** | **60** | **12** | **1** |  | 16 | 1 | **Infiltrating Ductal Adenocarcinoma** | **Well** |
| **39** | **1** | **74** | **Pancreatic cancer** | **37.56** | **T3N1M0** | **3** | **1** | **Ⅲ** | **55** | **9** | **1** |  | 33 | 1 | **Adenocarcinoma** | **Poorly** |
| **40** | **2** | **47** | **Pancreatic cancer** | **323.30** | **T2N1M0** | **2** | **1** | **Ⅲ** | **50** | **8** | **1** |  | 7 | 1 | **Infiltrating Ductal Adenocarcinoma** | **Poorly** |
| **41** | **1** | **57** | **Pancreatic cancer** | **891.20** | **T4N0M1** | **4** | **0** | **ⅣB** | **60** | **9** | **1** |  | 14 | 1 | **Infiltrating Ductal Adenocarcinoma** | **Moderately** |
| **42** | **1** | **75** | **Pancreatic cancer** | **1000.00** | **T2N2M1** | **2** | **2** | **ⅣB** | **55** | **9** | **1** |  | 26 | 0 | **Adenocarcinoma** | **Poorly** |
| **43** | **2** | **71** | **Pancreatic cancer** | **127.70** | **T2N1M0** | **2** | **1** | **Ⅲ** | **40** | **6** | **1** |  | 22 | 1 | **Adenocarcinoma** | **Moderately** |
| **44** | **2** | **64** | **Pancreatic cancer** | **985.60** | **T2N1M0** | **2** | **1** | **Ⅲ** | **25** | **8** | **1** |  | 17 | 1 | **Adenocarcinoma** | **Moderately** |
| **45** | **1** | **68** | **Pancreatic cancer** | **193.90** | **T1cN1M0** | **1** | **1** | **2** | **30** | **4** | **2** | **4** | 23 | 1 | **Adenocarcinoma** | **Moderately** |
| **46** | **1** | **72** | **Pancreatic cancer** | **184.60** | **T2N2M0** | **2** | **2** | **3** | **20** | **4** | **2** | **4** | 22 | 1 | **Infiltrating Ductal Adenocarcinoma** | **Moderately** |
| **47** | **1** | **55** | **Pancreatic cancer** | **241.55** | **T3N1M0** | **3** | **1** | **3** | **30** | **2** | **2** | **2** | 8 | 1 | **Infiltrating Ductal Adenocarcinoma** | **Poorly** |
| **48** | **2** | **43** | **Pancreatic cancer** | **152.10** | **T2N0M0** | **2** | **0** | **2** | **25** | **2** | **2** |  | 14 | 1 | **Infiltrating Ductal Adenocarcinoma** | **Poorly** |
| **49** | **1** | **65** | **Pancreatic cancer** | **52.00** | **T2N0M0** | **2** | **0** | **2** | **25** | **3** | **2** | **3** | 39 | 0 | **Infiltrating Ductal Adenocarcinoma** | **Poorly** |
| **50** | **1** | **53** | **Pancreatic cancer** | **25.20** | **T1cN1M0** | **1** | **1** | **2** | **8** | **3** | **2** | **4** | 31 | 1 | **Infiltrating Ductal Adenocarcinoma** | **Well** |
| **51** | **1** | **61** | **Pancreatic cancer** | **90.22** | **T2N0M0** | **2** | **0** | **2** | **25** | **2** | **2** | **3** | 16 | 1 | **Infiltrating Ductal Adenocarcinoma** | **Moderately** |
| **52** | **1** | **75** | **Pancreatic cancer** | **10.30** | **T4N2M1** | **4** | **2** | **4b** | **35** | **4** | **2** | **4** | 12 | 1 | **Adenocarcinoma** | **Poorly** |
| **53** | **1** | **64** | **Pancreatic cancer** | **17.10** | **T2N0M0** | **2** | **0** | **2** | **35** | **4** | **2** | **2** | 19 | 1 | **Adenocarcinoma** | **Moderately** |
| **54** | **1** | **66** | **Pancreatic cancer** | **82.70** | **T2N0M0** | **2** | **0** | **2** | **10** | **3** | **2** | **4** | 18 | 1 | **Adenocarcinoma** | **Moderately** |
| **55** | **2** | **63** | **Pancreatic cancer** | **85.60** | **T2N0M0** | **2** | **0** | **2** | **20** | **2** | **2** | **3** | 40 | 0 | **Infiltrating Ductal Adenocarcinoma** | **Poorly** |
| **56** | **2** | **56** | **Pancreatic cancer** | **38.10** | **T2N0M0** | **2** | **0** | **2** | **5** | **4** | **2** | **2** | 16 | 1 | **Adenocarcinoma** | **Moderately** |
| **57** | **1** | **58** | **Pancreatic cancer** | **33.55** | **T2N0M0** | **2** | **0** | **2** | **40** | **4** | **2** | **3** | 31 | 0 | **Infiltrating Ductal Adenocarcinoma** | **Poorly** |
| **58** | **2** | **75** | **Pancreatic cancer** | **36.73** | **T2N2M0** | **2** | **0** | **2** | **5** | **3** | **2** | **4** | 26 | 0 | **Infiltrating Ductal Adenocarcinoma** | **Poorly** |
| **59** | **2** | **59** | **Pancreatic cancer** | **65.40** | **T2N1M0** | **2** | **1** | **3** | **20** | **4** | **2** |  | 16 | 1 | **Infiltrating Ductal Adenocarcinoma** | **Moderately** |
| **60** | **2** | **65** | **Pancreatic cancer** | **42.10** | **T2N1M0** | **2** | **1** | **3** | **30** | **4** | **2** |  | 20 | 1 | **Infiltrating Ductal Adenocarcinoma** | **Moderately** |
| **61** | **2** | **49** | **Pancreatic cancer** | **22.30** | **T1cN1M0** | **1** | **1** | **2** | **40** | **2** | **2** |  | 35 | 1 | **Infiltrating Ductal Adenocarcinoma** | **Moderately** |
| **62** | **2** | **33** | **Pancreatic cancer** | **79.44** | **T2N2M0** | **2** | **2** | **3** | **15** | **3** | **2** |  | 19 | 1 | **Adenocarcinoma** | **Moderately** |
| **63** | **1** | **65** | **Pancreatic cancer** | **22.85** | **T1cN1M0** | **1** | **1** | **2** | **30** | **3** | **2** | **3** | 33 | 0 | **Infiltrating Ductal Adenocarcinoma** | **Poorly** |
| **64** | **1** | **54** | **Pancreatic cancer** | **46.00** | **T2N1M0** | **2** | **1** | **3** | **5** | **4** | **2** |  | 26 | 1 | **Adenocarcinoma** | **Moderately** |
| **65** | **1** | **57** | **Pancreatic cancer** | **354.20** | **T2N0M0** | **2** | **0** | **2** | **20** | **3** | **2** |  | 42 | 0 | **Adenocarcinoma** | **Moderately** |
| **66** | **2** | **60** | **Pancreatic cancer** | **14.11** | **T2N2M0** | **2** | **2** | **3** | **20** | **4** | **2** |  | 16 | 1 | **Infiltrating Ductal Adenocarcinoma** | **Well** |
| **67** | **1** | **53** | **Pancreatic cancer** | **83.06** | **T1cN1M0** | **1** | **1** | **2** | **15** | **3** | **2** |  | 38 | 0 | **Adenocarcinoma** | **Moderately** |
| **68** | **1** | **62** | **Pancreatic cancer** | **2.35** | **T2N0M0** | **2** | **0** | **2** | **25** | **2** | **2** |  | 36 | 1 | **Adenocarcinoma** | **Moderately** |
| **69** | **1** | **59** | **Pancreatic cancer** | **251.10** | **T2N1M0** | **2** | **1** | **3** | **40** | **3** | **2** |  | 25 | 1 | **Infiltrating Ductal Adenocarcinoma** | **Poorly** |
| **70** | **1** | **47** | **Pancreatic cancer** | **124.60** | **T2N0M0** | **2** | **0** | **2** | **35** | **3** | **2** |  | 42 | 0 | **Adenocarcinoma** | **Poorly** |
| **71** | **1** | **40** | **Pancreatic cancer** | **194.40** | **T2N2M0** | **2** | **2** | **3** | **60** | **4** | **2** |  | 28 | 1 | **Infiltrating Ductal Adenocarcinoma** | **Moderately** |
